# Tolerance engineering in* Deinococcus geothermalis* by heterologous efflux pumps

**DOI:** 10.1038/s41598-021-83339-1

**Published:** 2021-02-19

**Authors:** Erika Boulant, Emmanuelle Cambon, Julia Vergalli, Rémi Bernard, Fabienne Neulat-Ripoll, Flora Nolent, Olivier Gorgé, Maria Girleanu, Anne-Laure Favier, Jean-Paul Leonetti, Jean Michel Bolla

**Affiliations:** 1 Aix Marseille Univ, INSERM, SSA, IRBA, MCT, Marseille, France; 2 Deinove, Cap Sigma/ZAC Euromédecine II, Grabels, France; 3 Institut de Recherche Biomédicale des Armées, Département Microbiologie et Maladies Infectieuses, Unité Bactériologie, Brétigny-sur-Orge, France; 4 Institut de Recherche Biomédicale des Armées, Département des Plateformes et Recherche Technologique, Unité Imagerie, Brétigny-sur-Orge, France; 5 Present Address: Vilmorin SA, Centre de Recherche de La Costière, Ledenon, France; 6 Present Address: Institut de Recherche en Infectiologie de Montpellier, UMR 9004-CNRS/UM, Montpellier, France

**Keywords:** Expression systems, Industrial microbiology, Molecular engineering, Biochemistry, Biological techniques, Biotechnology, Microbiology

## Abstract

Producing industrially significant compounds with more environmentally friendly represents a challenging task. The large-scale production of an exogenous molecule in a host microfactory can quickly cause toxic effects, forcing the cell to inhibit production to survive. The key point to counter these toxic effects is to promote a gain of tolerance in the host, for instance, by inducing a constant flux of the neo-synthetized compound out of the producing cells. Efflux pumps are membrane proteins that constitute the most powerful mechanism to release molecules out of cells. We propose here a new biological model, *Deinococcus geothermalis*, organism known for its ability to survive hostile environment; with the aim of coupling the promising industrial potential of this species with that of heterologous efflux pumps to promote engineering tolerance. In this study, clones of *D. geothermalis* containing various genes encoding chromosomal heterologous efflux pumps were generated. Resistant recombinants were selected using antibiotic susceptibility tests to screen promising candidates. We then developed a method to determine the efflux efficiency of the best candidate, which contains the gene encoding the MdfA of *Salmonella enterica serovar* Choleraesuis. We observe 1.6 times more compound in the external medium of the hit recombinant than that of the WT at early incubation time. The data presented here will contribute to better understanding of the parameters required for efficient production in *D. geothermalis*.

## Introduction

*Deinococcus geothermalis*, initially isolated from hot springs^1^, is an aerobic, non-pathogenic, non-sporulating, non-flagellar, pinkish-red bacterium that typically exists as dyads and tetrads. Despite a positive response to Gram’s stain, the envelope of *Deinococci* has an unusual organization and consists of multilayers approaching the didermes^2–4^. Because *Deinococci* present characteristics attributed to both Gram-positive (peptidoglycan^5–9^) and Gram-negative (outer membrane^10–12^ and fatty acid^12–14^) bacteria, the phylogenetic classification of *Deinococci* in the bacterial kingdom remains controversial^5,13,15,16^.

*D. geothermalis* is increasingly being investigated and used for various applications due to its surprising ability to withstand environments considered hostile to other bacteria (ionizing radiation, desiccation and raised temperature)^1,17,18^. Currently, *D. geothermalis* is the best candidate for the bioremediation of soils contaminated with radionuclide wastes^1,17,19,20^ or xenobiotics^21^. This bacterium was recently chosen as a new model organism to determine the impact of exposure in space or Martian conditions on a bacterial biofilm^22^. *D. geothermalis* is a thermophilic organism with promising applications in human therapy due to its ability to produce UV-resistant extremolytes as well as carotenoids with significant antioxidant potential^23,24^.

For all these reasons, *D. geothermalis* has been selected and used as a cellular microfactory by the biotechnology company Deinove specialized in the synthesis of innovative compounds from extremophilic organisms. This collaborative work between industrial and academic research was born out of an industrial need to take the next step in the level of production of compounds. Thanks to the development of chromosomal cloning tools developed on *Deinococcus* spp.^25^ (WO2015092013), the company dispose of organisms able to produce bioethanol from sugar polymer-containing biomass (WO2009063079, WO2010094665A2, WO 2010/130806, US20140356923A1), terpenes and terpenoids (WO 2015/189428), astaxanthin (WO2017212030A1) and phytoene (WO2018109194A1) compounds, all having an application in the bioenergy, pharmaceutical, nutritional and cosmetic fields.

In addition, bacteria from *Deinococcus spp*. have been the subject of other industrial applications, where specific enzymes of *D. geothermalis* or *D. radiodurans* are used for the production of molecules, such as α-arbutin (KR20110028169, Kyung Hee University—Industry Cooperation Group), nucleoside triphosphates and ribonucleic acid (WO2017176963 and WO2019075167, Greenlight Biosciences Inc.).

The large-scale production of an endogenous or exogenous compound in a host microfactory can cause detrimental consequences. Indeed, a high level of synthesis and intracellular accumulation of a compound can interfere with physiological processes and damage the cell envelope, leading to negative feedback regulation of the host metabolism and/or cell death^26–28^. Host tolerance to exogenous compounds is key to producing high value-added compounds on a large scale. The concept of tolerance engineering first described by Dunlop and its collaborators^29^ is the engineering of biological mechanisms that allow the bacterial host to become tolerant to the compound produced.

Efflux pumps are a well-known mechanism for the extrusion of toxic compounds out of the cell and largely contribute to the multiresistance of bacteria to antibiotics^30–32^. In the context of bioproduction, they are used as biological tools to release toxic compounds out of the producing cell^29,33,34^. For that reason, these membrane protein complexes have been integrated into our model organism genetically designed for production, *D. geothermalis*. For the two cloning strategies (overexpression of a homologous or heterologous efflux pump), we favored the cloning of heterologous efflux pumps because of the insufficient data available on *D. geothermalis* efflux pumps^35^, with the aim of obtaining *D. geothermalis* recombinants with functional efflux pumps.

This study, genes encoding highly diverse heterologous efflux pumps were transferred into *D. geothermalis*; these efflux pumps belonging to five different families, were selected from various bacterial species, and are known to have different substrates. The susceptibility of the recombinants to various antibiotics was tested in order to pre-select candidates with a possibly high efflux capability; one recombinant was selected, and its efflux ability was further characterized using innovative fluorescent assays adapted for the first time to *D. geothermalis*. The identified candidate expresses a functional efflux pump, MdfA of *Salmonella enterica serovar* Choleraesuis. This pump increases the amount of extracellular compounds and thus decreases the intracellular accumulation of compounds. This study illustrates one of the numerous possible applications of efflux pumps in biotechnology for bioproduction.

## Results

### Successful cloning of heterologous efflux pumps in *Deinococcus geothermalis* chromosome

Twenty-seven efflux pump genes were cloned in *D. geothermalis* by homologous recombination at the same locus on the chromosome under the control of a constitutive promoter optimized for expression*.* The cloning of at least one representative of the different families of bacterial efflux pumps has been considered (MFS-Major Facilitator Superfamily, SMR-Small Multidrug Resistance family, MATE- Multidrug and Toxic Compounds Extrusion family, ABC- ATP-Binding Cassette family and Transporter). The heterologous pumps were selected according to their substrate selectivity, with a focus on compounds of industrial interest or for their multispecies presence. The chromosomal insertion was expected to be more stable than a replicative plasmid, and the use of a constitutive promoter has many advantages for future industrial process: no inducer compound needed during bioproduction, reducing the cost for the industry, and the efflux pump will be continually expressed. The heterologous pumps were selected with the aim of having a large panel of various efflux pump exhibiting a large diversity of substrate (multi-drug efflux pumps), and expressed in various species (see the efflux pump characteristics in Table 1). Among the 27 different selected efflux pump genes, 20 were successfully cloned, and their characteristics are described in Table 1. Integration and DNA sequences encoding efflux pumps were validated for each recombinant by colony PCR (Supplementary Figure S1) and sequencing.

**Table 1:** Heterologous efflux pump genes selected for chromosomal cloning in *D. geothermalis* DSM11300.

ID	NCBI reference sequence protein (CDS region in nucleotide sequence)	Characteristics of the efflux pump	Donor organism	Source	Size (bp/aa)	Point mutation(s)
**MFS**—**major facilitator superfamily**						
P1	GI : 383796854 (CP003416.1:c651732-650491)	Major Facilitator Superfamily, transporter MdtM	*Salmonella enterica subsp. enterica s*erovar Heidelberg str. B182	DSM No.: 17420	1242 bp/413 aa	/
P6	GI : 24982814 (NC_002947.4:1543197-1544441)	Multispecies MFS transporter	*Pseudomonas putida KT2440*	DSM No.: 6125	1245 bp/414 aa	TTT-1203-TTC (F-401-F)
P8	GI : 374354065 (NC_016832.1:c2084953-2083721)	Multidrug translocase mdfA	*Salmonella enterica subsp. enterica *serovar Typhi	DSM No.: 10062	1233 bp/410 aa	
P11	WP_011588684.1 (NC_008260.1:c1599672-1598455)	Bcr/CflA family multidrug resistance transporter	*Alcanivorax borkumensis SK2*	DSM No.: 11573	1218 bp/405 aa	
P13	NP_455648.1 (CP019194.1:1971911-1973124)	Multidrug resistance protein MdtG	*Salmonella enterica subsp. enterica *serovar Typhi	DSM No.: 10062	1215 bp/404 aa	/
P15	GI : 24985252 (NC_002947.4:4076656-4077843)	Multidrug resistance transporter, Bcr/CflA family	*Pseudomonas putida KT2440*	DSM No.: 6125	1188 bp/395 aa	CAA-476-CGA (Q-159-R)
P16	GI : 24984944 (NC_002947.4:3739558-3740796)	Multidrug resistance transporter, Bcr/CflA family	*Pseudomonas putida KT2440*	DSM No.: 6125	1239 bp/412 aa	/
P19	GI :148505197 (NC_009525.1:c1408650-1407391)	Multispecies: multidrug resistance efflux protein	*Mycobacterium tuberculosis H37Ra*	ATCC 25177D-5	1260 bp/419 aa	
P20	APW08100.1 (CP019194.1:4818131-4819003)	Inner membrane transport protein YieO	*Salmonella enterica subsp. enterica* serovar Typhi	DSM No.: 10062	873 bp/290 aa	/
P22	GI :148504778 (NC_009525.1:947362-948621)	Multispecies: putative conserved integral membrane transport protein	*Mycobacterium tuberculosis H37Ra*	ATCC 25177D-5	1260 bp/419 aa	
P23	ABX21941.1 (CP000880.1:1995580-1996812)	Multidrug transporter MdfA	*Salmonella enterica subsp. enterica *serovar Choleraesuis	DSM No.: 9386	1233 bp/410 aa	CAT-6-CAG (H-2-Q);
						AAC-9-AAT (N-3-N);
						TCG-726-TCA (S-242-S)
**SMR**—**small multidrug resistance family**						
P24	GI : 26988433 (NC_002947.4:c1897073-1896756)	Quaternary ammonium compound-resistance protein SugE	*Pseudomonas putida KT2440*	DSM No.: 6125	318 bp/105 aa	/
P25	WP_011589815.1 (AM286690.1:c2885459-2885130)	QacE family quaternary ammonium compound efflux	*Alcanivorax borkumensis SK2*	DSM No.: 11573	330 bp/109 aa	/
P26	GI : 553895183 (NC_022594.1:c1678585-1678256)	Multidrug/spermidine efflux SMR transporter subunit MdtI	*Pseudomonas aeruginosa PAO1-VE13*	DSM No.: 22644	330 bp/109 aa	/
P27	GI : 553900134 (NC_022594.1:3986875-3987288)	Multispecies: Small Multidrug Resistance family protein	*Pseudomonas aeruginosa PAO1-VE13*	DSM No.: 22644	414 bp/137 aa	
P28	GI : 553899956 (NC_022594.1:5606098-5606430)	Multispecies: Small Multidrug Resistance family protein	*Pseudomonas aeruginosa PAO1-VE13*	DSM No.: 22644	333 bp/110 aa	/
P30	GI : 26991608 (NC_002947.4:5610586-5610918)	QacE family quaternary ammonium compound efflux	*Pseudomonas putida KT2440*	DSM No.: 6125	333 bp/110 aa	/
P31	GI : 15598461 (NC_002516.2:3653138-3653452)	Quaternary ammonium compound-resistance protein SugE	*Pseudomonas aeruginosa PAO1*	DSM No.: 22644	315 bp/104 aa	/
P34	GI : 553897854 (NC_022594.1:c1678947-1678579)	Multidrug/spermidine efflux SMR transporter subunit MdtJ	*Pseudomonas aeruginosa PAO1-VE13*	DSM No.: 22644	369 bp/122 aa	/
P36	GI : 383800511 (NC_017623.1:4669621-4670481)	EamA-like transporter family	*Salmonella enterica subsp. enterica *serovar Heidelberg str. B182	DSM No.: 17420	861 bp/286 aa	GCG-66-GCA (A-33-A);
						GCG-330-GCA (A-110-A);
						TCC-525-TCA (S-175-S);
						GCG-762-GCA (S-254-S)
**MATE**—**multidrug and toxic compounds extrusion family**						
P41	WP_011588223.1 (AM286690.1:1052518-1053798)	Cell surface polysaccharide transporter	*Alcanivorax borkumensis SK2*	DSM No.: 11573	1281 bp/426 aa	/
P43	GI : 383796609 (NC_017623.1:360980-362305)	MATE family efflux transporter DinF	*Salmonella enterica subsp. enterica *serovar Heidelberg str. B182	DSM No.: 17420	1326 bp/441 aa	
P44	GI : 383796342 (NC_017623.1:51027-52277)	WzxE protein, also named lipid III flippase WzxE	*Salmonella enterica subsp. enterica *serovar Heidelberg str. B182	DSM No.: 17420	1251 bp/416 aa	/
P48	AGY69778.1 (NC_022594.1:2466535-2467944)	MviN-like family protein	*Pseudomonas aeruginosa PAO1-VE13*	DSM No.: 22644	1410 bp/469 aa	GTT-130-CTT (V-44-L)
**ABC**—**ATP-binding cassette family**						
P56	WP_000551246.1 (NC_003197.2:1070438-1072186)	MsbA	*Salmonella enterica subsp. enterica* serovar Typhimurium str. LT2	DSM No.: 17058	1749 bp/582 aa	
P58	WP_003906469.1 (NC_009525.1:1407391-1408650)	Phosphate ABC transporter ATP-binding protein, also named PstB	*Mycobacterium tuberculosis H37Ra*	ATCC 25177D-5	831 bp/276 aa	/
**Transporter**						
P64	WP_003859640.1 (NC_003450.3:2344432-2346051)	Predicted permease; succinate exporter	*Corynebacterium glutamicum K051*	ATCC 13032	1620 bp/539 aa	/

Genes highlighted in grey have not been successfully cloned. ‘/’: no mutations.

### Selection of recombinants with antibiotic tolerance enhancement

To select recombinant candidates with a better antibiotic tolerance than the WT strain and consequently with a possibly high efflux capability, we turned to the determination of their susceptibility to antibiotics. The classical minimum inhibitory concentration (MIC) assay in microplates was adapted for the *D. geothermalis* strains. These susceptibility assays were used as a high-throughput selection to refocus further analyses on candidates with any gain of tolerance to antibiotics compared to the wild-type strain (WT). The MICs of at least one antibiotic per family used clinically to date were determined for each recombinant. The MICs obtained with the various recombinants were compared to the MICs of the WT strain. A minimum fourfold increase in MIC values was considered to indicate a gain of resistance. The recombinant clones that were less susceptible to various antibiotics than the parental host are listed in Table 2. The other recombinants with similar susceptibilities to the WT (and thus not selected for the remainder of the study) are presented in Supplementary Table S1.

**Table 2:**
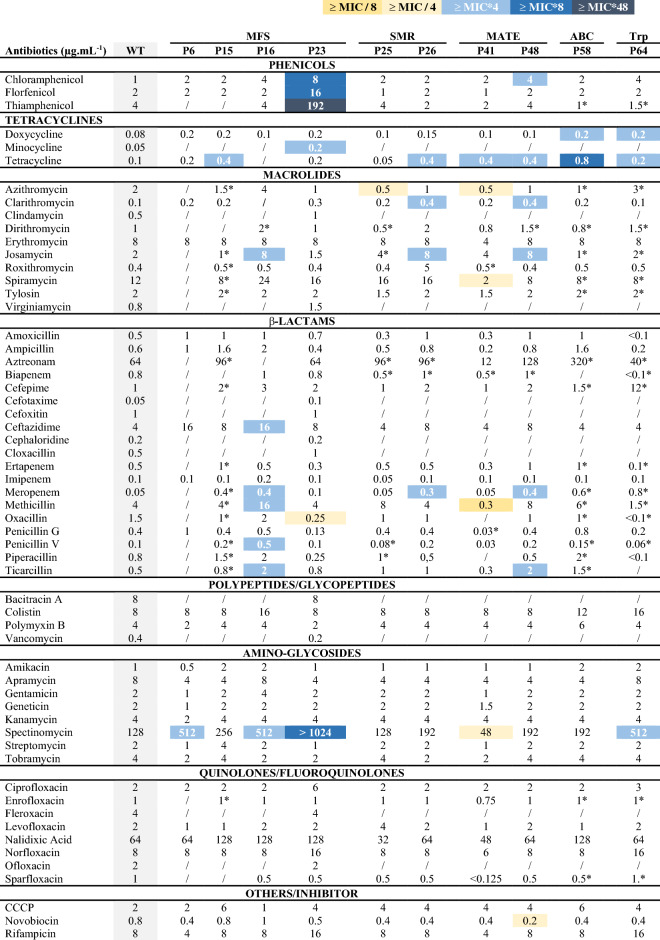
Median values of MICs (µg mL^−1^) of various antibiotics determined on WT and recombinant *D. geothermalis* for various efflux pump genes.

*D. geothermalis* recombinants are named P6, P15, P16, P23, P25, P26, P41, P48, P58 and P64. MFS, Major Facilitator Superfamily; SMR, Small Multidrug Resistance proteins; MATE, Multidrug and Toxic Compounds Extrusion; ABC, ATP-Binding Cassette Transporter; RND, Resistance-Nodulation Cell Division; Trp, Transporter. ‘P’, recombinants. ‘/’, not determined. (*) Tests performed in duplicate; all other tests were performed in at least three biological replicates, and median were presented. The MIC values with differences between recombinants and WT strain are indicated by colours according to the colour scale displayed above the table.

The MIC of P25 did not show any pronounced increase compare to the WT ones, except a slight decrease of tolerance for the azithromycin. For this reason, this recombinant strain was chosen as a negative control in this study. Conversely, the MIC values of the recombinants P6, P15, P16, P26, P41, P48, P58 and P64 for different antibiotics were up to 4 times higher than those of the WT and were therefore considered for further analysis. Among the clones harboring efflux pumps of the MFS family, P6 showed a lower susceptibility to spectinomycin, P15 showed a lower susceptibility to tetracycline, and P16 showed a lower susceptibility to josamycin, ceftazidim, meropenem, methicillin, penicillin V, ticarcillin and spectinomycin compared to the WT strain. Concerning the SMR family, P26 presented a lower susceptibility to tetracyclin, clarithromycin, josamycin and meropenem and P25 presented a slight decrease of tolerance for azithromycin. Concerning the MATE family, P41 presented a lower susceptibility to tetracycline and a higher sensitivity to other antibiotics (azithromycin, spiramycin, methicillin and spectinomycin). P48 was less susceptible to chloramphenicol, tetracycline, clarithromycin, josamycin, meropenem and ticarcillin and presented a higher sensitivity to novobiocine. P58, with an efflux pump from the ABC family, showed a lower susceptibility to tetracycline and doxycycline, while the recombinant P64, which carries an efflux pump predicted to be involved in succinate transport, showed a lower susceptibility to tetracycline, doxycycline and spectinomycin. Surprisingly, we did not find any correlation between the predicted and/or demonstrated activity of the pumps in their natural environment and their activity when expressed in *Deinococcus*. In addition, the specificity of the molecular class of antibiotics could not be highlighted in any of the cases mentioned above.

P23, with an MdfA efflux pump from *Salmonella enterica* subsp. *enterica* serovar Choleraesuis, showed a 8-, 8-, 48-, 8- and 4-times increase in tolerance for chloramphenicol, florfenicol, thiamphenicol, spectinomycin and minocycline, respectively; and a slight decrease in tolerance for the oxacillin compared to the WT. In this case, an increased tolerance to three antibiotics from the phenicol family might suggest a specificity, at least partially. Moreover, the very high increase in the MIC of thiamphenicol on P23 (48-times compared to the WT) evidenced the efficient activity of the encoded pump in *Deinococcus*. Consequently, we decided to focus our study on P23.

### Recombinants are sustainable

#### Genomic sequencing

The whole genome sequences of P23 and P25 (used as a negative control) were analyzed in order to verify (1) the presence of the mega-amplicon only in the desired region, (2) the absence of mutations, deletions or insertions in the genome, and (3) the absence of mutations in the phenicol target, the 50S subunit of ribosomal RNA. Reading depths of 20 X (99.6% coverage) and 22 X (99.7% coverage) for the chromosome (NC_008025.1), 1.3 X (46% coverage) and 11.7 X (97.8% coverage) for the plasmid pDGEO01 (NC_008010.2) and 20.9 X (98.5% coverage) and 24.7 X (98.1% coverage) for the plasmid pGEO02 (NC_009939.1) were obtained for P23 and P25, respectively. We did not find any genotypic difference with the genome of the WT, even when looking more specifically at the rRNA genes of *D. geothermalis* (data not shown)*.*

#### Transmission electron microscopy

**Figure 1:**
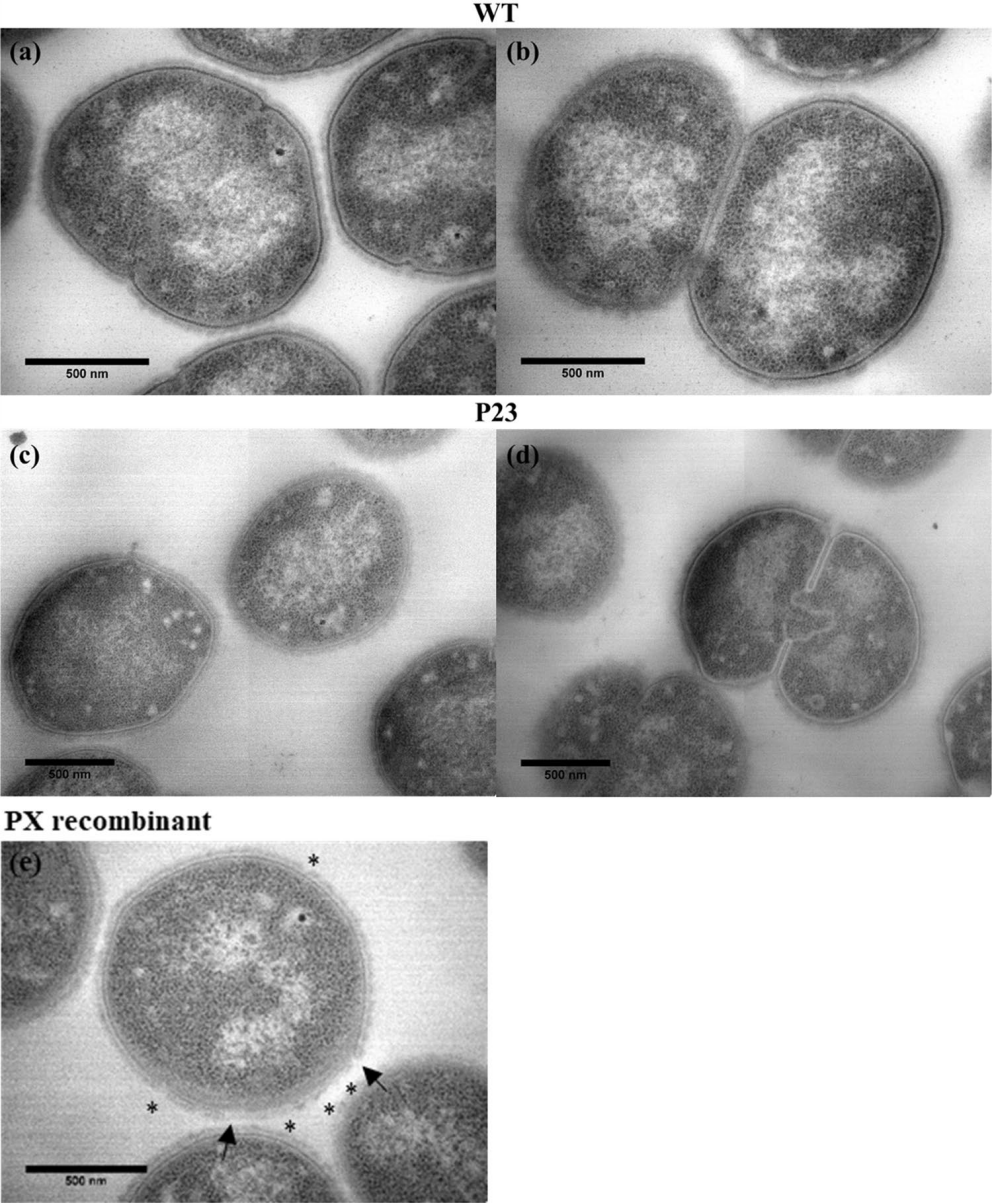
Electron micrograph images of *D. geothermalis*: Individual cells (**a**,**c**,**e**) and cells during division (**b**,**d**) of the wild-type strain DSM11300 (WT; **a**,**b**), P23 recombinant strain (**c**,**d**) and PX recombinant (**e**). PX recombinant’s image (**e**) shows a noticeable destabilization of different parts of the envelope. The stars represent a destabilization of the carbohydrate layer and the arrows of the lipid bilayer. The recombinant PX shows a strongly truncated insertion of a gene coding for the efflux pump (improper pump insertion).

The integrity of the envelope of P23 was observed by TEM imaging (Fig. 1). The images did not show noticeable differences in the envelope between the WT and P23 strains, regardless of whether the cells were dividing. More images of the two strains were recorded (Supplementary Figure S2) in order to assess whether the integrity of the membrane is maintained within the majority of cells of the bacterial population. These images were compared with images of another recombinant with a strongly truncated insertion of an efflux pump coding gene (Fig. 1). We observed an obvious destabilization of the different parts of the envelope, with a destabilization of the carbohydrate coat and the lipid bilayer.

### Real time efflux of Hoechst 33342

To investigate the contribution of the MdfA efflux pump a multidrug/proton antiporter that derives its energy from the transmembrane proton gradient from *S.* Choleraesuis (MdfA_*SC*_, P23) to the susceptibility results, the efflux capacity was evaluated by measuring the accumulation of a fluorescent probe, Hoechst 33342. This dye is one of the most widely used fluorescent probes to measure the efflux capacity of bacterial strains. The real-time efflux protocol previously developed by Bonhert and coworkers^36^ was adapted for *D. geothermalis* to compare the accumulation of Hoechst fluorescence in the WT, P23 and P25 (used as a negative control) strains over time in the absence or presence of either glucose and ATP (to provide energy to the cells and thus promote efflux) or CCCP (to collapse the energy-driving force of the efflux pump) at 42 °C (Supplementary Figure S3). For each strain, the accumulation of fluorescence of Hoechst alone (in blue) was lower than in the presence of CCCP (in red). Similarly, the accumulation of Hoechst fluorescence in the presence of glucose and ATP (in green) was obviously lower than without glucose and ATP.

The fluorescence level of Hoechst accumulated in cells was not significantly different between the three strains (Student’s t-test, p-value > 0.05, data not shown). In this context, we can assume that the efflux pump cloned in P23 does not expel Hoechst 33342.

### MdfA_***SC***_, a functional efflux pump in ***D. geothermalis***

To further study the ability of the MdfA_*SC*_ efflux pump cloned in P23 to expel molecules belonging to the phenicol family, measurements of fluorescence accumulation of thiamphenicol were carried out. Thiamphenicol was chosen because it showed the highest increase in MIC in P23 compared to WT (see Table 2). It was not possible to measure the intracellular accumulation of thiamphenicol in this context for several reasons: (1) conventional *D. geothermalis* lysis techniques result in a loss of fluorescence of the compound; and (2) the lifetime of thiamphenicol exposed to a temperature of 42 °C greatly reduces, decreasing its fluorescence intensity over time. For this purpose, an experimental assay was designed based on the techniques set up in our laboratory^37–39^ to measure the real-time amount of thiamphenicol in the extracellular environment during the incubation period. This content of thiamphenicol consisted of the amount that did not accumulate in cells plus the amount expelled by the efflux pump.

**Figure 2:**
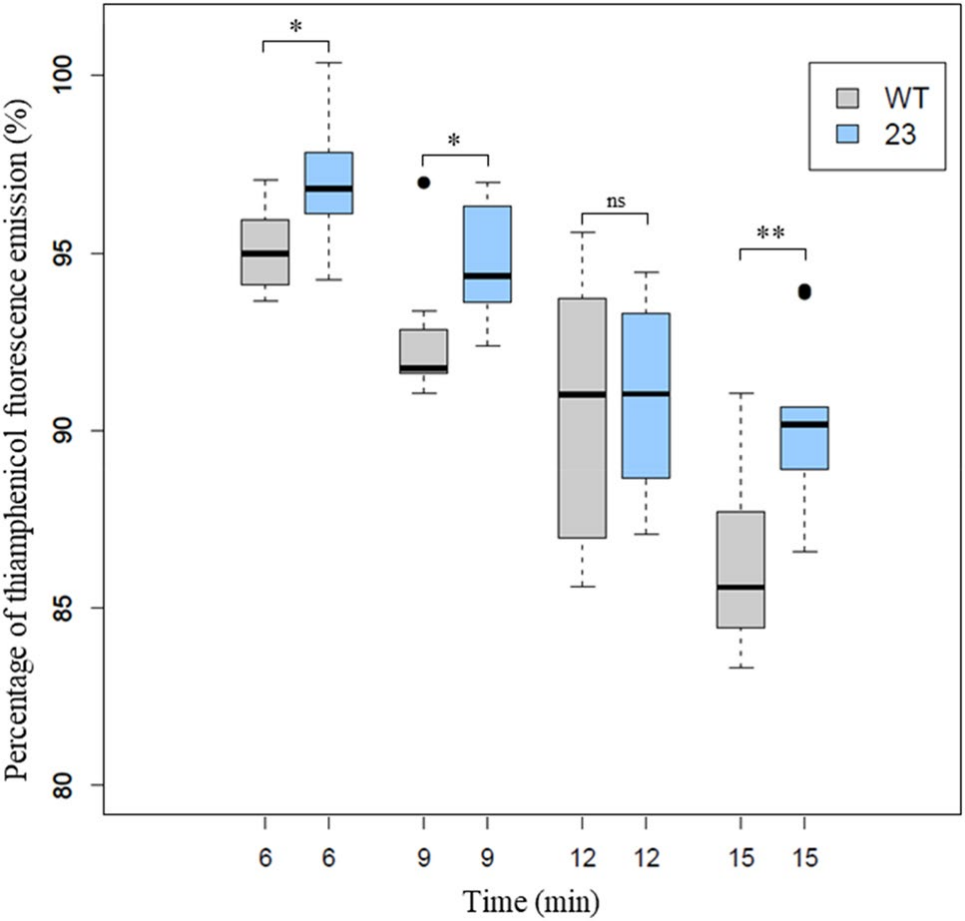
Percentages of fluorescence intensities of thiamphenicol (zoomed to 80–100%) in the extracellular environment measured during the incubation of WT (grey boxes) and P23 (blue boxes) strains. The box-and-whisker plot shows the medians (“center” lines), 25th (lower section) and 75th (upper section) percentiles (interquartile range; box edges) and the span of the data for each sample (whiskers). ANOVA and Tukey’s post-hoc tests were performed to determine differences between the two strains (6 min, n = 17; 9 min, n = 18; 12 min, n = 14; 15 min, n = 18). ***p < 0.001; **p < 0.01; *p < 0.05. Data standardization was performed by the Shapiro–Wilk test, and homogeneity of variances was checked by the Fligner-Killeen test. *Ns* not significant. The significant difference between the two strains for the entire incubation kinetics was extremely significant p < 0.001 (ANOVA, n = 67, Cohen’s f = 0.51). Four independent experiments were performed (with technical triplicate).

A time course study of thiamphenicol fluorescence intensity was performed with 100 µg mL^−1^ at 42 °C with *D. geothermalis* WT and P23 recombinant strains (Fig. 2 and Supplementary Figure S4). The percent fluorescence levels of thiamphenicol measured in the extracellular environment were converted to box and whisker plots^40^ for a graphical representation of the statistical data (Fig. 2).

Overall, the fluorescence emission level of thiamphenicol in the extracellular environment was significantly higher (p < 0.001, ANOVA, n = 67, Cohen’s f = 0.5) in P23 (blue boxes) than in WT (gray boxes). The percentage of thiamphenicol fluorescence in the extracellular environment of the P23 strain was significantly higher than that in the WT strain at 6 min (*p* < 0.05), 9 min (*p* < 0.05) and 15 min (*p* < 0.01). The coefficient of determination of the trend curve, r^2^, based on fluorescence emission averages of thiamphenicol in the extracellular environment over time, was 0.995 for the WT and 0.9808 for the P23 recombinant (data not shown). The plot of the fitted slopes of the two strains highlights the difference between P23 and WT strains at the early time of incubation (Supplementary Figure S5). After 6 min of incubation, the fluorescence of thiamphenicol in the extracellular environment of P23 was 1.6-fold (95% confidence interval 1.2–2.0) greater than that in WT. A higher signal strength of thiamphenicol in the extracellular environment implies a lower amount of thiamphenicol inside the cells and a higher efflux capacity. These results are concordant, in this context, with a functional efflux pump MdfA_*SC*_ in the P23 recombinant concomitant with concentrations 1.6-fold higher than that WT in the extracellular medium.

## Discussion

The aim of this study was to demonstrate that cloning heterologous efflux pumps could result in a decrease in the intracellular amount of a toxic agent and consequently an increased tolerance. This work reoriented on a public health problem, the role of efflux pumps in multi-drug resistance, towards a promising industrial use.

The effectiveness of chromosomal integration of heterologous sequences in *D. geothermalis* has been validated; 75% of genes were integrated out of the 27 different genes belonging to various species selected, regardless of the size of the fragment. However, further studies are needed to correlate the donor organism of the gene and the success of the expression of a functional protein in *D. geothermalis*. It would thus be interesting to extend the selection to other Gram-negative species, such as *Acinetobacter baumannii*, *Alcanivorax borkumensis*^41^, *Klebsiella pneumoniae*, *Enterobacter aerogenes*, and *Campylobacter jejuni*, and Gram-positive organisms, such as *Staphylococcus aureus* and *Bacillus subtilis*, which are all known to produce efficient efflux pumps.

The antibiotic susceptibility results (MICs) of the WT strain are interesting because they allowed us to observe the natural resistance profile of *D. geothermalis* for a wide range of antibiotics. Sensitivity to phenicols, cyclins, β-lactams (except for the monobactam subfamily), aminoglycosides, and macrolides was observed, as well as low resistance to fluoroquinolones and polypeptides (except glycopeptides). Overall, the resistance profile of the WT strain was similar to those of common Gram-positive bacteria. In addition, the sensitivity of *D. geothermalis* to beta-lactams suggests that this species does not encode beta-lactamases. These susceptibility results highlighted that this technique can be used as an initial tool for the selection of resistant or sensitive *D. geothermalis* recombinants.

Among the recombinants with low susceptibility to antibiotics, P23, which expresses an MdfA efflux pump, was selected based on its selective tolerance to phenicol compounds. The MdfA efflux pumps from *Salmonella* Choleraesuis have been found to confer resistance to tetracycline, chloramphenicol, norfloxacin, and doxorubicin in a strain deleted for the *acrB* gene and overexpressing the homologous protein MdfA^42^. In our study, P23 did not show any noticeable increase in tolerance to tetracycline, norfloxacin (the MIC was twice that of WT) or doxorubicin (data not shown) during susceptibility testing. The WT strain of *D. geothermalis* had an MIC of 8 µg/mL for norfloxacin. According to CASFM (Comité de l’Antibiogramme de la Société Française de Microbiologie) recommendations, an *Enterobacteriaceae* species with an MIC strictly greater than 1 µg.mL^-1^ is resistant to norfloxacin (http://www.sfmmicrobiologie.org/UserFiles/files/casfm/CASFMV2_SEPTEMBRE2018.pdf). The data obtained with norfloxacin and tetracycline led to the same conclusion. These results highlight the importance of considering the host organism during antibiotic resistance studies.

P23 showed a gain of tolerance to florfenicol, thiamphenicol and spectinomycin. The efflux pump FloR (MFS) is known to specifically transport phenicols in *E. coli* ^39^ and to confer a much higher resistance to thiamphenicol than to chloramphenicol or florfenicol. The same phenotypes were observed in this study with P23 expressing MdfA_*SC*_. The expression of a heterologous efflux pump in another species distant from the parent organism, such as *D. geothermalis*, makes it possible to identify other efflux pump substrates that may not be detected in the donor organism due to its natural resistance. The opposite can also be true: some compounds known to be substrates of a given efflux pump may not be found as such due to the intrinsic resistance of the host species.

To test that the decreased susceptibilities of P23 were concordant with a functional efflux pump, we turned to the most commonly used fluorescent dye, Hoechst 33342, and measured its efflux capacity. However, this dye did not seem to be a substrate of the efflux pump cloned in P23 under the conditions used. This analysis underlines the fact that Hoechst 33342 may not be suitable for all bacteria.

Therefore, an assay previously developed in the literature and in our laboratory^37–39^ was set up to measure the ability of P23 to transport a fluorescent antibiotic, thiamphenicol. To our knowledge, thiamphenicol has not been used as a fluorescent agent to measure the efflux capacity of a bacterium. Since the optimum growth temperature of *D. geothermalis* is 48 °C, the limited stability of the antibiotic under these conditions was considered, and a temperature of 45 °C was used during the susceptibility assays. Moreover, for the transport assays the incubation of bacteria in the presence of thiamphenicol, experiments were performed at 42 °C to ensure the thiamphenicol fluorescence was relatively stable. Only a slight decrease in the fluorescence signal of 4% was observed after 12 min of incubation at 42 °C (data not shown). The monitoring of efflux of thiamphenicol developed herein could probably be used for other bacteria. The data presented here show that fluorescence methods can be applied to *D. geothermalis* at a temperature of 42 °C. However, to get even closer to the optimal growth temperature of this organism, it would be interesting to perform the same tests with a fluorescent marker that would not be impacted by a temperature of 48 °C. It can be assumed that the efflux capacity observed at 42 °C could be much higher at 48 °C, the optimal growth temperature for this bacterium, which has a more efficient metabolism (such as protein synthesis, export and assembly) at this temperature. Conversely, one must also consider that a heterologous efflux pump from an organism with an optimal growth temperature of 37 °C could be less stable or less efficient at 48 °C. Consequently, it could be more efficient to express efflux pumps from organisms with a growth temperature closer to 48 °C in *D. geothermalis*. As an example, efflux pumps from *Campylobacter jejuni* (optimal growth temperature of 42 °C) could represent a good alternative.

Typical fluorometric assays allow the determination of the efflux capacity by measuring the intracellular concentration of a fluorescent compound after the complete lysis of bacteria and by comparison with in a strain deleted for the efflux pump^38^. In this study, the extracellular amount of the fluorescent antibiotic was measured because efficient lysis of this robust bacterium is difficult to achieve. This technique was challenging due to the large amount of the compound in the extracellular environment compared to the intracellular environment. Indeed, according to the work of Zhou and coworkers^39^, if the efflux capacity of the MdfA pump was not high enough, no difference in the amount of thiamphenicol in the supernatant between the WT and P23 would be observed. A decrease in all fluorescence signals over time was observed during the experiment, that can be explained by the depletion of the cells (which were not re-energized). Indeed, the expression of efflux pumps and other physiological mechanisms require energy; this explains the progressive decrease in their efflux capacity. In this case, to observe the efflux capacity of MdfA*sc*, it was necessary to perform tests in a buffer, but in an industrial context, the bacteria are fed with a constant supply of nutrients. Only precise measurements and a high number of replicates ensured that the fluorescence intensity was significantly higher in the extracellular environment of P23 compared to that of WT, with a ratio of 1.6 at 6 min of incubation. If we apply our method of calculations to the results obtained by Zhou and collaborators in a Δ*tolC* deletion mutant, the linezolid concentration ratio is 1.5 times lower in the mutant strain than in the WT strain^39^.

The global strategy adopted in this study could be extended to RND-type efflux pumps, which are known to expel a great diversity of substrates^43^. Nevertheless, further studies are needed to determine the feasibility of cloning genes coding for these protein complexes in a phylogenetically distant bacterium with an atypical envelope^5^. Moreover, expression of an RND efflux pump can lead to a high energy cost as well as destabilization of the bacterial envelope.

In conclusion, this study adapts various methods to *D. geothermalis* and demonstrates that the strategy of using heterologous efflux pumps (1) is achievable in the atypical and non-pathogenic organism *D. geothermalis*, (2) possibly reduces the toxicity of high value-added compounds, and (3) can be transferred to non-pathogenic cellular microfactories such as modified *D. geothermalis* for industrial production.

## Materials and methods

### Bacterial strains and growth conditions

The bacterial strain *Deinococcus geothermalis* DSM11300 and its derivatives used in the present study are listed in Table 1. Bacteria were grown in PGY broth/agar (PGYA) (10 g L^−1^ peptone, 10 g L^−1^ glucose, 5 g L^−1^ yeast extract and 14 g L^−1^ agar) at 45 °C under aerobic conditions in hermetic jars with 4.5% (v/v) MilliQ sterile water to maintain the moisture ratios necessary for the optimal growth of *D. geothermalis*. Bleomycin was supplemented when required to a final concentration of 12 µg mL^−1^. Bleomycin was chosen because *D. geothermalis* is sensitive to this compound and because its structure is very different from those of the antibiotics used in this study. The bacterial cells were stored at − 80 °C with 20% (v/v) glycerol.

### Chromosomal cloning of heterologous efflux pumps

Several genes encoding heterologous efflux pumps were inserted into the chromosome of *D. geothermalis* DSM11300 between Dgeo_1606 (ABF45901.1, upstream gene) and Dgeo_1605 (ABF45900.1, downstream gene) by homologous recombination. The heterologous genes were cloned under the control of a promoter derived from *D. radiodurans* pGroESL^44,45^ For that purpose, 3 parts of the constructs (upstream, heterologous and downstream parts) were amplified by PCR using the primers listed in Supplementary Table S2. The upstream and downstream parts, common to all constructs, were amplified using the DNA extracted from a DSM11300 mutant previously constructed at Deinove company (unpublished data), whereas the heterologous regions were amplified using the DNA listed Table 1 as templates. The upstream region consisted of the chromosomal upstream region associated with the promoter derived from the *D. radiodurans* pGroESL. The downstream part contained a codon-optimized *D. geothermalis* bleomycin resistance cassette^46^ associated with the chromosomal downstream region.

The amplicons were purified and assembled with NEBuilder HiFi DNA assembly Master Mix (NEB). The final amplification product of the assembly reactions using primers oEG1345_F and oEG1365_R was analyzed on the Microfluidic Capillary Electrophoresis Systems (LabChip GX, PerkinElmer). The DNA fragments were then transformed by a CaCl_2_-dependent technique in DSM1300 cells^19^, and the transformants were selected on PGYA supplemented with bleomycin. The correct insertion on the chromosome was double-checked by PCR on colony (Supplementary Figure S1) using the two primer pairs oEC1059_F and oEC332_R (Supplementary Table S2) and sequenced with Sanger technology. All the primers used are referenced in the Supplementary Table S2.

### Chromosomal sequencing

*DNA extraction.* Strains were cultured as previously described. DNA was extracted from each strain using the bacterial DNA extraction kit DNAseasy Blood and Tissue (Qiagen) according to the manufacturer’s instructions. Genomic DNA was quantified using a QuBit (Qubit 2.0 fluorometer, Invitrogen, Thermo Fisher Scientific) with a Qubit dsDNA BR Assay kit (Thermo Fisher Scientific). Only high-quality DNA samples (OD_260/280 nm_ 1.8 ~ 2.0, > 6 μg) were used for sequencing.

#### Sequencing, assembly, and annotation

To construct libraries (200 to 300-bp-long), extracted DNAs were sheared on a M220 focused ultrasonicator (Covaris) with the recommended settings. The libraries were then prepared with the NEBNext Ultra DNA Kit (New England Biolabs) and NEBNext multiplexed oligonucleotides (NEBNext Multiplex Oligos for Illumina). Whole-genome sequencing was performed using the Illumina Nextseq platform (Illumina Inc.) by generating 2 × 150 bp paired-end libraries using the NextSeq 500 mid-output v2.5 kit (Illumina Inc.) following the manufacturer’s instructions. Bioinformatic analysis was performed using the following software packages: FastQCv.0.11.9^47^, Cutadaptv1.18^48^, BWAv0.7.17^49^ and SAMtoolsv1.19^50^. Visualization of the results was performed using Geneious R11 software (Biomatters Inc.). Expression and resistance cassettes of P23 and P25 strains, developed at DEINOVE, are available upon request to academic groups.

### Transmission electron microscopy analysis

Samples were fixed with 2.5% (v/v) glutaraldehyde (EMS) in cacodylate buffer (0.1 M, pH 7.4, 2% sucrose, with CaCl_2_ and MgCl_2_ (Merck Millipore)) overnight at 4 °C. After washing, samples were postfixed with 1% (v/v) osmium tetroxide in cacodylate buffer for 1 h at room temperature under a chemical hood and covered with aluminum foil. Then, samples were stained for 1 h at 4 °C with 2% (v/v) uranyl acetate and further dehydrated at different ethanol concentrations. Samples were embedded in Epon LX112 (Inland Europe) resin and subjected to polymerization at 60 °C for 48 h. Ultrathin Sects. 100 nm thick were cut with the ultramicrotome Reichert Ultracut S (Leica Microsystems) and placed onto 300 mesh copper grids. Sections were then double stained with 2% uranyl acetate and lead citrate (EMS). High-resolution transmission electron microscopy was performed with a CM10 microscope operating at 100 kV and equipped with a CCD Erlanghsen 1000 Gatan camera. No filtering procedure was applied to the images.

### Chemicals

The following compounds were solubilized in 100% DMSO: thiamphenicol, virginiamycin, roxithromycin, dirithromycin, azithromycin, spiramycin, josamycin, tylosin, cefaloridine, trimethoprim, and carbonyl cyanide m-chlorophenyl hydazone (CCCP). Chloramphenicol, florfenicol, tetracycline, and azthreonam were solubilized in 50% DMSO (v/v). Nalidixic acid, norfloxacin, ofloxacin, fleroxacin, sparfloxacin, and enrofloxacin were prepared in distilled water with 0.75% (v/v) NaOH. All other compounds were solubilized in sterile distilled water.

### Susceptibility testing

The MICs were determined in 96-well microplates by the microdilution method (without agitation) in accordance with the recommendations of the Comité de l’Antibiogramme de la Société Française de Microbiologie. The standard broth dilution method was performed with an inoculum of 2.10^5^ CFU/mL in 200 µL of PGY medium containing two-fold serial dilutions of each antibiotic. The microplate was coated with a sterile permeable membrane (Air-O-Seal; 4titude) that allows good gas exchange and humidity between the bacteria in the medium and the jar atmosphere. The microplates were incubated in hermetic jars filled with 4.5% (v/v) MilliQ sterile water. MIC values were read after 24 h of incubation at 45 °C by reading the OD_600nm_ with an Infinite M200 pro (Tecan). Experiments were carried out in biological triplicate and the resulting medians are presented.

### Measurements of Hoechst 33342 efflux

#### Preparation of bacterial suspension for real-time efflux (RTE) assays

A colony was inoculated in 5 mL of PGY broth in a 50 mL Falcon tube and incubated for 24 h at 45 °C with shaking (250 rpm). The day of the experiment, precultures were inoculated at 1:50 in fresh PGY broth. After 6 h of growth (corresponding to an OD_600nm_ of approximately 0.8), the cultures were centrifuged (4,000 × g, 20 min) at room temperature. Pellets were washed three times and resuspended in 50 mM PPB buffer (potassium phosphate buffer 50 M K_2_HPO_4_ containing 5 mM MgSO_4_, pH 7.2) to an OD_600nm_ of 2.5.

#### Spectrofluorimetric measurements

The fluorescence measurements were performed in a 96-well black half-well microplate (Greiner). 8 mL of each bacterial suspension was deposited with 5 µL of 2.5 µM Hoechst 33342 (Life Technologies) without or with 10 µL of 50 mM glucose and 5 µL of 1 mM ATP or 10 µM CCCP. The emission fluorescence peak of Hoechst 33342 was measured at approximately 420 nm (+ /- 20 nm) with an excitation wavelength of 370 nm (+ /- 9 nm) using a spectrofluorometer preheated at 42 °C (Infinite M200 Pro, Tecan).

### Measurements of thiamphenicol fluorescence emission in the extracellular environment

This protocol was adapted from^40,49–51^.

#### Preparation of bacterial suspension

A colony was inoculated in 5 mL of PGY broth in a 50 mL Falcon tube and incubated for 24 h at 45 °C with shaking at 250 rpm. The volume of liquid culture should not exceed 10% of the flask volume (to ensure enough oxygenation). On the day of the experiment, precultures were diluted in fresh PGY broth at 1:50. After approximately 6 h of growth (in the middle of the exponential phase of growth, corresponding to an OD_600nm_ of approximately 0.8), the culture was centrifuged (4000×*g*, 20 min, room temperature). Pellets were resuspended in NaPi-Mg buffer (50 mM sodium phosphate buffer NaH_2_PO_4_, containing 10 mM MgCl_2_, 0.5 mM ATP and 0.001% Tween 20, pH 7.2) to an OD_600nm_ of 7.6.

#### Accumulation assay

Before starting the assay, bacteria were preincubated at 42 °C for 15 min to allow the bacteria to recover under good physiological conditions. The fluorescence assay was performed in glass tubes (Pyrex) to avoid antibiotic adhesion to the surface of the plastic tubes. A total of 4.5 mL of each bacterial suspension (OD_600nm_ of 6.9) was incubated at 42 °C with 0.5 mL of 100 µg L^−1^ thiamphenicol in a final volume of 5 mL. During the accumulation assay, 250 µL of each sample was removed at various time points (0 min, and every 3 min for 12 min) and centrifuged at 6500×*g* for 5 min at room temperature. The supernatants were immediately recovered. Considering the time required for the centrifugation, the time point 0 min corresponded to 6 min for the accumulation kinetics. Bacterial suspensions incubated without antibiotics and antibiotics incubated without bacteria were used as controls. All accumulation assays were performed in triplicate.

#### Spectrofluorimetric measurements

The supernatants were loaded into a 96-well black half-well microplate, and the thiamphenicol fluorescence emission was recorded within the range of 280–340 nm (emission peak at approximately 293 nm) at an excitation wavelength of 231 nm using a spectrofluorometer (Infinite Pro, Tecan).

#### Data processing

During the accumulation assay, 100% thiamphenicol fluorescence was determined based on the fluorescence measurement of thiamphenicol incubated without bacteria, and the negative control (0%) was determined from the fluorescence of the buffer incubated without bacteria or thiamphenicol. The correlation between the fluorescence emission of thiamphenicol and its concentration was validated by performing a calibration curve (Supplementary Figure S4): to determine the reliability of the correlation between the fluorescence signal and the antibiotic concentration, the fluorescence emission signals of thiamphenicol at various concentrations, including the concentration used during the assay, were recorded. The reliability of this linear correlation was checked by the determination of Pearson’s correlation coefficient r^2^. Thiamphenicol fluorescence concentrations (Fig. 2) were obtained from n = 4 independent experiments. Statistical analysis was performed using the computing environment R (R Development Core Team, 2005). ANOVAs with Tukey’s post hoc tests were used to determine differences between WT and P23 strains shown in Fig. 2. P values between 0.01 and 0.5 were considered significant (*), P values between 0.001 and 0.01 were considered very significant (**), and P values < 0.001 were considered extremely significant (***). The data normality and homoscedasticity were checked by the respective Shapiro–Wilk and Fligner–Killeen tests. The ratio between the amount of thiamphenicol fluorescence remaining in the supernatant after incubation with the WT and the P23 clone (at the first time of incubation (6 min) was highlighted in Supplementary Figure S5 and can be calculated as follows: ratio = 100 − fluorescence in the supernatant of the wt/100 − fluorescence in the supernatant of P23.

## Supplementary Information

Supplementary Information

## References

[1] Ferreira, A. C. *et al.* Deinococcus geothermalis sp. nov. and Deinococcus murrayi sp. nov., two extremely radiation-resistant and slightly thermophilic species from hot springs. *Int. J. Syst. Bacteriol.* **47**, 939–947 (1997). 10.1099/00207713-47-4-939 9336890

[2] Rothfuss H (2006). Involvement of the S-layer proteins Hpi and SlpA in the maintenance of cell envelope integrity in Deinococcus radiodurans R1. Microbiology.

[3] Misra, C. S., Basu, B. & Apte, S. K. Surface (S)-layer proteins of Deinococcus radiodurans and their utility as vehicles for surface localization of functional proteins. *Biochimica et Biophysica Acta (BBA) - Biomembranes* **1848**, 3181–3187 (2015). 10.1016/j.bbamem.2015.09.021 26450150

[4] Yu, J. *et al.* A tamB homolog is involved in maintenance of cell envelope integrity and stress resistance of Deinococcus radiodurans. *Sci. Rep.* **7**, (2017). 10.1038/srep45929 PMC5382914 28383523

[5] Gupta RS (2011). Origin of diderm (Gram-negative) bacteria: antibiotic selection pressure rather than endosymbiosis likely led to the evolution of bacterial cells with two membranes. Antonie Van Leeuwenhoek.

[6] Murray, R. G. E. The Family Deinococcaceae. in *The Prokaryotes* 3732–3744 (Springer, New York, NY, 1992). 10.1007/978-1-4757-2191-1_42.

[7] Gupta RS (1998). Protein phylogenies and signature sequences: A reappraisal of evolutionary relationships among archaebacteria, eubacteria, and eukaryotes. Microbiol. Mol. Biol. Rev..

[8] Gupta RS, Mukhtar T, Singh B (1999). Evolutionary relationships among photosynthetic prokaryotes (Heliobacterium chlorum, Chloroflexus aurantiacus, cyanobacteria, Chlorobium tepidum and proteobacteria): implications regarding the origin of photosynthesis. Mol. Microbiol..

[9] Quintela, J. C., Portillo, F. G., Pittenauer, E., Allmaier, G. & Pedro, M. A. de. Peptidoglycan fine structure of the radiotolerant bacterium deinococcus radiodurans sark. *J. Bacteriol.* **181**, 334–337 (1999). 10.1128/jb.181.1.334-337.1999 PMC103566 9864347

[10] Thornley MJ, Horne RW, Glauert AM (1965). The fine structure of Micrococcus radiodurans. Arch Mikrobiol.

[11] Lancy P, Murray RG (1978). The envelope of Micrococcus radiodurans: Isolation, purification, and preliminary analysis of the wall layers. Can. J. Microbiol..

[12] Murray, R. G. E. Bergey’s manual of systematic bacteriology. 1035–1043 (1986).

[13] Brooks BW (1980). Red-Pigmented micrococci: a basis for taxonomy. Int. J. Syst. Evol. Microbiol..

[14] Counsell TJ, Murray RGE (1986). Polar lipid profiles of the genus deinococcus. Int. J. Syst. Evol. Microbiol..

[15] Woese CR (1987). Bacterial evolution. Microbiol. Rev..

[16] Roberts E, Sethi A, Montoya J, Woese CR, Luthey-Schulten Z (2008). Molecular signatures of ribosomal evolution. PNAS.

[17] Liedert C, Peltola M, Bernhardt J, Neubauer P, Salkinoja-Salonen M (2012). Physiology of resistant deinococcus geothermalis bacterium aerobically cultivated in low-manganese medium. J. Bacteriol..

[18] Ranawat P, Rawat S (2017). Radiation resistance in thermophiles: mechanisms and applications. World J. Microbiol. Biotechnol..

[19] Brim H, Venkateswaran A, Kostandarithes HM, Fredrickson JK, Daly MJ (2003). Engineering Deinococcus geothermalis for bioremediation of high-temperature radioactive waste environments. Appl. Environ. Microbiol..

[20] Makarova, K. S. *et al.* Deinococcus geothermalis: The Pool of Extreme Radiation Resistance Genes Shrinks. *PLoS ONE* **2**, (2007). 10.1371/journal.pone.0000955 PMC1978522 17895995

[21] Kongpol A, Kato J, Vangnai AS (2008). Isolation and characterization of Deinococcus geothermalis T27, a slightly thermophilic and organic solvent-tolerant bacterium able to survive in the presence of high concentrations of ethyl acetate. FEMS Microbiol. Lett..

[22] Frösler J, Panitz C, Wingender J, Flemming H-C, Rettberg P (2017). Survival of Deinococcus geothermalis in biofilms under desiccation and simulated space and martian conditions. Astrobiology.

[23] Singh, O. V. & Gabani, P. Extremophiles: radiation resistance microbial reserves and therapeutic implications. *J. Appl. Microbiol.* **110**, 851–861 (2011). 10.1111/j.1365-2672.2011.04971.x 21332616

[24] Farci, D., Slavov, C., Tramontano, E. & Piano, D. The S-layer Protein DR_2577 binds deinoxanthin and under desiccation conditions protects against UV-radiation in deinococcus radiodurans. *Front. Microbiol.* **7**, (2016). 10.3389/fmicb.2016.00155 PMC4754619 26909071

[25] Gerber E (2015). Deinococcus as new chassis for industrial biotechnology: Biology, physiology and tools. J. Appl. Microbiol..

[26] Clomburg, J. M., Crumbley, A. M. & Gonzalez, R. Industrial biomanufacturing: The future of chemical production. *Science* **355**, aag0804 (2017). 10.1126/science.aag0804 28059717

[27] Chen X (2018). DCEO biotechnology: tools to design, construct, evaluate, and optimize the metabolic pathway for biosynthesis of chemicals. Chem. Rev..

[28] Langevin AM, Dunlop MJ (2018). Stress introduction rate alters the benefit of AcrAB-TolC efflux pumps. J. Bacteriol..

[29] Dunlop MJ (2011). Engineering microbial biofuel tolerance and export using efflux pumps. Mol. Syst. Biol..

[30] Nikaido H, Pagès J-M (2012). Broad specificity efflux pumps and their role in multidrug resistance of gram negative bacteria. FEMS Microbiol. Rev..

[31] Li X-Z, Plésiat P, Nikaido H (2015). The challenge of efflux-mediated antibiotic resistance in gram-negative bacteria. Clin. Microbiol. Rev..

[32] Du D (2018). Multidrug efflux pumps: Structure, function and regulation. Nat. Rev. Microbiol..

[33] Mukhopadhyay A (2015). Tolerance engineering in bacteria for the production of advanced biofuels and chemicals. Trends Microbiol..

[34] Wu G (2016). Metabolic burden: Cornerstones in synthetic biology and metabolic engineering applications. Trends Biotechnol..

[35] Tian B (2010). Proteomic analysis of membrane proteins from a radioresistant and moderate thermophilic bacterium Deinococcus geothermalis. Mol. BioSyst..

[36] Bohnert JA (2016). Novel piperazine arylideneimidazolones inhibit the AcrAB-TolC pump in escherichia coli and simultaneously act as fluorescent membrane probes in a combined real-time influx and efflux assay. Antimicrob. Agents Chemother..

[37] Dumont E (2018). Antibiotics and efflux: combined spectrofluorimetry and mass spectrometry to evaluate the involvement of concentration and efflux activity in antibiotic intracellular accumulation. J. Antimicrob. Chemother..

[38] Vergalli J (2018). Spectrofluorimetric quantification of antibiotic drug concentration in bacterial cells for the characterization of translocation across bacterial membranes. Nat. Protoc..

[39] Zhou Y (2015). Thinking outside the ‘bug’: a unique assay to measure intracellular drug penetration in gram-negative bacteria. Anal. Chem..

[40] Tukey JW (1977). Some thoughts on clinical trials, especially problems of multiplicity. Science.

[41] Schneiker S (2006). Genome sequence of the ubiquitous hydrocarbon-degrading marine bacterium Alcanivorax borkumensis. Nat. Biotechnol..

[42] Nishino K, Latifi T, Groisman EA (2006). Virulence and drug resistance roles of multidrug efflux systems of Salmonella enterica serovar Typhimurium. Mol. Microbiol..

[43] Nikaido H (2018). RND transporters in the living world. Res. Microbiol..

[44] Meima R, Rothfuss HM, Gewin L, Lidstrom ME (2001). Promoter cloning in the radioresistant bacterium Deinococcus radiodurans. J. Bacteriol..

[45] Schmid AK, Lidstrom ME (2002). Involvement of two putative alternative sigma factors in stress response of the radioresistant bacterium deinococcus radiodurans. J. Bacteriol..

[46] Brouns SJJ (2005). Engineering a selectable marker for hyperthermophiles. J. Biol. Chem..

[47] Andrews. Babraham Bioinformatics - FastQC A Quality Control tool for High Throughput Sequence Data. https://www.bioinformatics.babraham.ac.uk/projects/fastqc/ (2010).

[48] Martin, M. Cutadapt removes adapter sequences from high-throughput sequencing reads. *EMBnet. J.* **17**, 10–12 (2011).

[49] Li H, Durbin R (2009). Fast and accurate short read alignment with Burrows-Wheeler transform. Bioinformatics.

[50] Li H (2009). The Sequence Alignment/Map format and SAMtools. Bioinformatics.

[51] Zhou Z (2015). CYP287A1 is a carotenoid 2-β-hydroxylase required for deinoxanthin biosynthesis in Deinococcus radiodurans R1. Appl. Microbiol. Biotechnol..

[52] Masi M, Réfregiers M, Pos KM, Pagès J-M (2017). Mechanisms of envelope permeability and antibiotic influx and efflux in Gram-negative bacteria. Nat. Microbiol..

[53] Masi, M., Dumont, E., Vergalli, J., Pajovic, J., Réfrégiers, M. & Pagès JM. Fluorescence enlightens RND pump activity and the intrabacterial concentration of antibiotics. *Res Microbiol.*10.1016/j.resmic.2017.11.005. 10.1016/j.resmic.2017.11.005 29208490

