# Supplementary material for: Tolerance engineering in Deinococcus geothermalis by heterologous efflux pumps
Source: Sci Rep. 2021 Feb 19;11:4280. doi: 10.1038/s41598-021-83339-1 (PMC7896070; doi:10.1038/s41598-021-83339-1)
Supplement: Supplementary file 1 — Supplementary Information [file 41598_2021_83339_MOESM1_ESM.pdf]

Supplementary material

Tolerance engineering in *Deinococcus geothermalis* by heterologous efflux pumps.

Erika Boulant[1,2], Emmanuelle Cambon[2], Julia Vergalli[1], Rémi Bernard[2], Fabienne Neulat-Ripoll[3], Flora Nolent[3], Olivier Gorgé[3], Maria Girleanu[4], Anne-Laure Favier[4], Jean-Paul Leonetti[2], and Jean-Michel Bolla[1*]

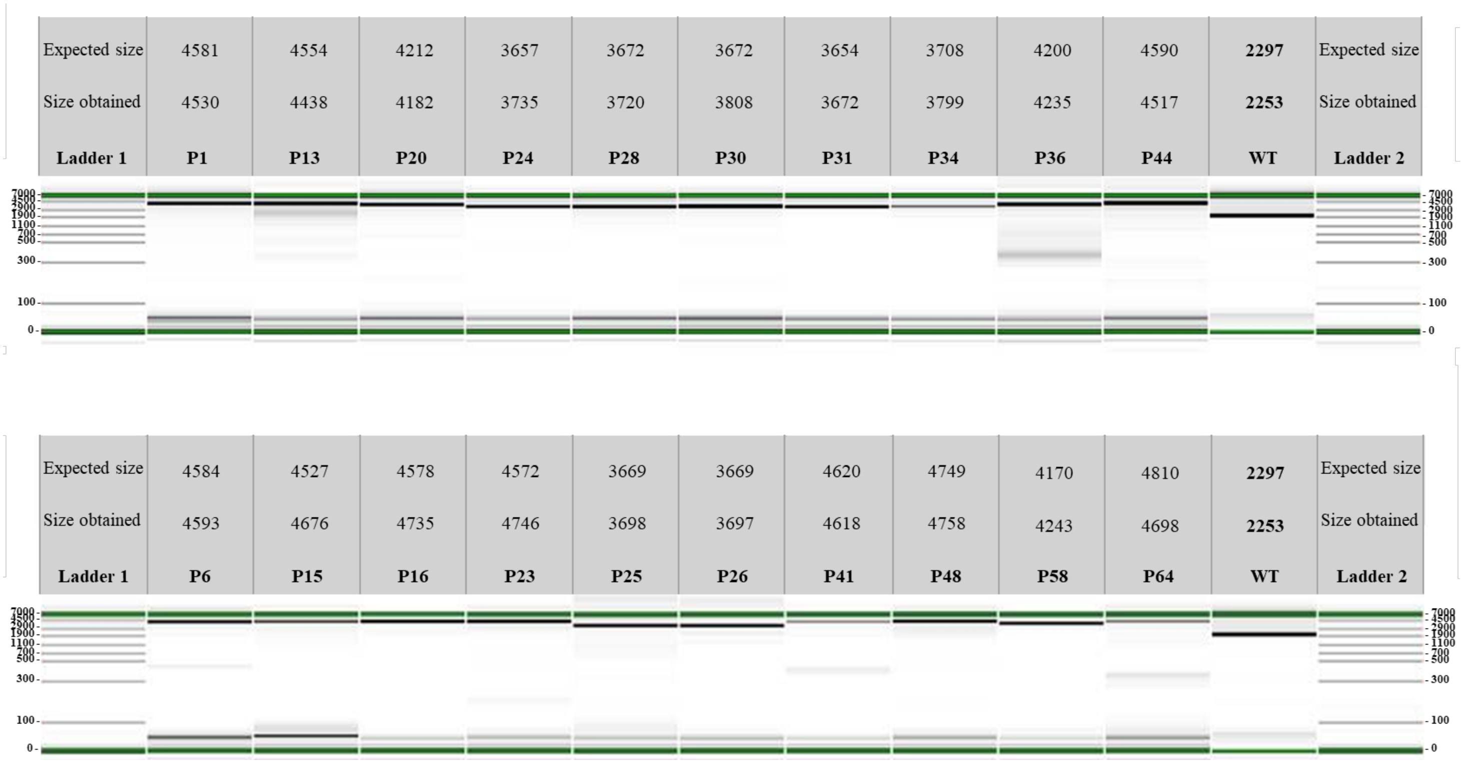

Supplementary Figure S1. Colony PCR to verify the presence of the efflux pump insert. The primers used are oEC1059_F and oEC332_R (Supplementary Table S2).

≥ MIC / 8 | ≥ MIC / 4 | ≥ MIC*4 | ≥ MIC*8 | ≥ MIC*48

		MFS			SMR						MATE
**Antibiotics (µg.mL^{-1})**	**WT**	**P1**	**P13**	**P20**	**P24**	**P28**	**P30**	**P31**	**P34**	**P36**	**P44**
**PHENICOLS**											
Chloramphenicol	1	1	2	1	1	1	2	1	2	1	2
Florfenicol	2	2	4	2	0.5	2	2	2	2	2	2
Thiamphenicol	4	/	/	/	/	/	/	/	/	/	/
**TETRACYCLINES**											
Doxycycline	0.08	0.05	0.1	0.1	0.1	0.1	0.1	0.1	0.1	0.1	0.1
Mynocycline	0.05	/	/	/	/	/	/	/	/	/	/
Tetracycline	0.1	0.05	0.2	0.1	0.1	0.2	0.2	0.2	0.2	0.2	0.2
**MACROLIDES**											
Azithromycin	2	/	/	/	/	/	/	/	/	/	/
Clarithromycin	0.1	0.05	0.2	0.05	0.05	0.15*	0.2	0.1	0.2	0.1*	0.2
Clindamycin	0.5	/	/	/	/	/	/	/	/	/	/
Dirithromycin	1	/	/	/	/	/	/	/	/	/	/
Erythromycin	8	6	8	8	4	8	8	8	8	8	8
Josamycin	2	/	/	/	/	/	/	/	/	/	/
Roxithromycin	0.4	/	/	/	/	/	/	/	/	/	/
Spiramycin	12	/	/	/	/	/	/	/	/	/	/
Tylosin	2	/	/	/	/	/	/	/	/	/	/
Virginiamycin	0.8	/	/	/	/	/	/	/	/	/	/
**BETA-LACTAMS**											
Amoxicillin	0.5	1	1	0.5	0.3	0.5	1	1	1	0.5	0.5
Ampicillin	0.6	1	2	1*	0.8	0.8	0.8	0.8	0.8	0.4	0.8
Aztreonam	64	/	/	/	/	/	/	/	/	/	/
Biapenem	0.8	/	/	/	/	/	/	/	/	/	/
Cefepime	1	/	/	/	/	/	/	/	/	/	/
Cefotaxime	0.05	/	/	/	/	/	/	/	/	/	/
Cefoxitin	1	/	/	/	/	/	/	/	/	/	/
Ceftazidime	4	8	16	8	4	8	8	8	8	4	4
Cephaloridine	0.2	/	/	/	/	/	/	/	/	/	/
Cloxacillin	0.5	/	/	/	/	/	/	/	/	/	/
Ertapenem	0.5	/	/	/	/	/	/	/	/	/	/
Imipenem	0.1	0.1	0.1	0.1	0.1	0.1	0.1	0.1	0.1	0.1	0.1
Meropenem	0.05	/	/	/	/	/	/	/	/	/	/
Methicillin	4	/	/	/	/	/	/	/	/	/	/
Oxacillin	1.5	/	/	/	/	/	/	/	/	/	/
Penicillin G	0.4	1	1	0.1	0.4	0.4	0.4	0.8	0.4	0.2	0.4
Penicillin V	0.1	/	/	/	/	/	/	/	/	/	/
Piperacillin	0.8	/	/	/	/	/	/	/	/	/	/
Ticarcillin	0.5	/	/	/	/	/	/	/	/	/	/
**POLYPEPTIDES/GLYCOPEPTIDES**											
Bacitracin A	8	/	/	/	/	/	/	/	/	/	/
Colistin	8	16	8	16	8	16	8	8	8	8	8
Polymyxin B	4	4	2	4	4	4	4	4	2	4	4
Vancomycin	0.4	/	/	/	/	/	/	/	/	/	/
**AMINO-GLYCOSIDES**											
Amikacin	1	1	0.5	1	1	2	1	1	1	1	1
Apramycin	8	8	4	8	8	8	4	8	8	4	8
Geneticin	2	2	1	2	2	2	2	4	2	2	2
Gentamicin	2	2	2	2	2	2	2	2	2	2	2
Kanamycin	4	4	2	4	4	4	4	4	4	4	4
Spectinomycin	128	32	384*	64	64	64	128	256	128	256	128*
Streptomycin	2	2	1	1	2	2	2	1	2	1	2
Tobramycin	4	4	2	2	4	4	4	4	4	2	2
**QUINOLONES**											
Ciprofloxacin	2	2	2	2	2	2	2	2	2	2	2
Enrofloxacin	1	/	/	/	/	/	/	/	/	/	/
Fleroxacin	4	/	/	/	/	/	/	/	/	/	/
Levofloxacin	2	1	2	1	2	2	2	2	2	2	2
Nalidixic Acid	64	64	128	64	32	64	64	64	64	64	64
Norfloxacin	8	8	8	8	8	8	8	8	8	8	8
Ofloxacin	2	/	/	/	/	/	/	/	/	/	/
Sparfloxacin	1	/	/	/	/	/	/	/	/	/	/
**OTHERS/INHIBITOR**											
CCCP	2	2	4	4	4	4	4	4	4	4	2
Novobiocin	0.8	0.4	0.3	0.4	0.2	0.3	0.2	0.2	0.2	0.4	0.2
Rifampicin	8	4	8	8	4	8	8	8	8	8	4

Supplementary Table S1. Median values of MICs (µg.mL^{-1}) of *D. geothermalis* recombinant strains that were not selected for the remainder of the study. *D. geothermalis* recombinants are named P1, P13, P20, P24, P28, P30, P31, P34, P36 and P44. MFS, Major Facilitator Superfamily; SMR, Small Multidrug Resistance Family; MATE, Multidrug and Toxic Compounds Extrusion; ABC, ATP-Binding Cassette Transporter; RND, Resistance-Nodulation Cell Division; Trp, Transporter. " P ", recombinants. " / ", not determined. (*) Tests were performed in duplicate, all other tests were performed in at least three biological replicates, and medians were presented. MIC values with differences between recombinants and WT strain are indicated by colours according to the colour scale displayed above the table.

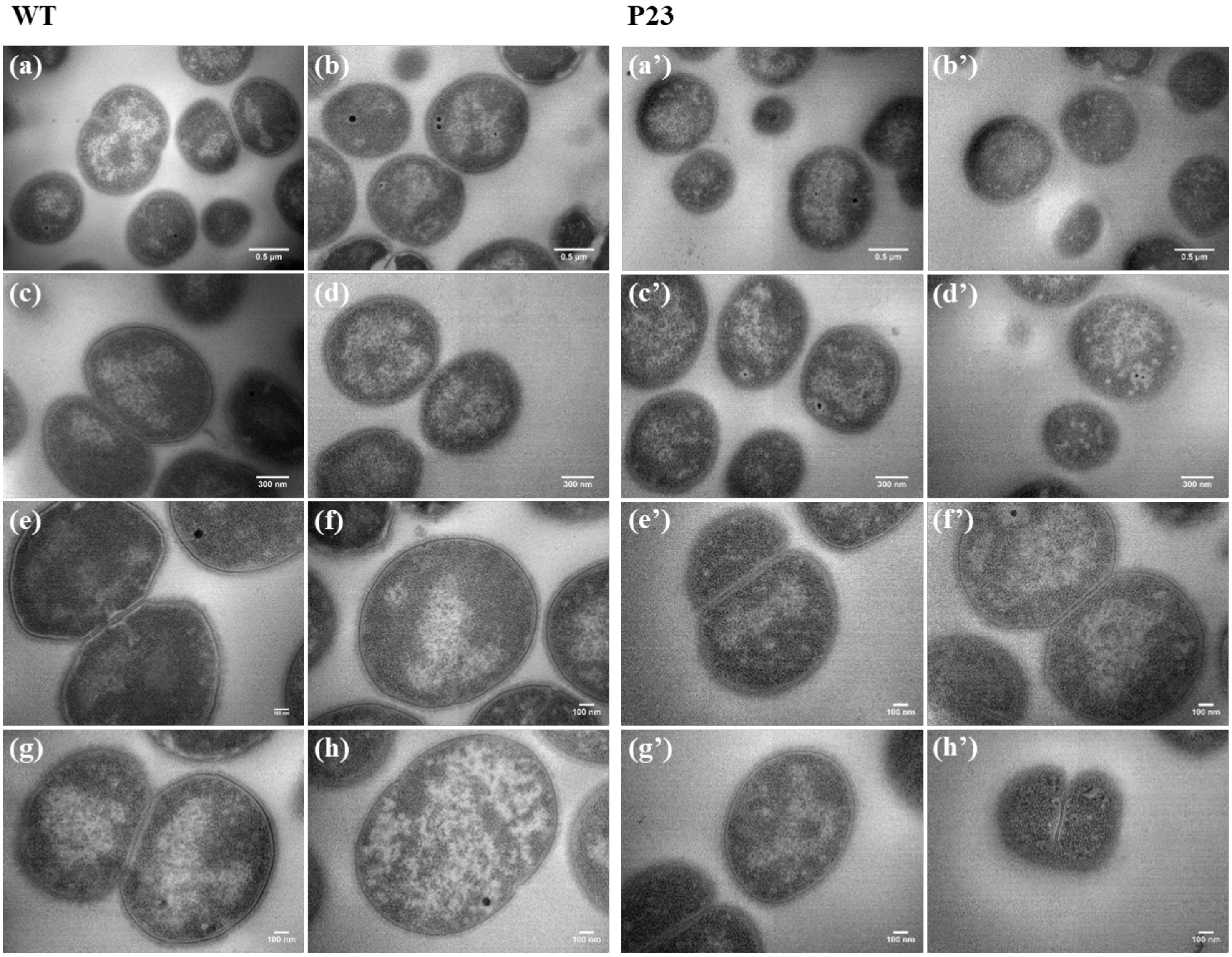

Supplementary Figure S2. Electron micrography imagery of *D. geothermalis*. Both individual cells and cells during division of (a-j) the wild type strain DSM11300 (WT) and (a'-j') the P23 recombinant strain.

Supplementary Table S2. Primers used for the cloning of heterologous efflux pumps.

Primers	Sequences (5' to 3')	Used for
	**Upstream amplicon**	
oEG1344_F	GAGCTCGGTACCCGGGGATCCGTTCACGCACCACATAGCCCAGAC	
oEC043_R	GTGGGGTCCTCCTGTGAGG	
	**Heterologous amplicon (efflux pump)**	
oEC050_F	CTCACAGGAGGACCCCACATGCAACGGATTATTCAGTTTTTCTCC	P1
oEC051_R	GCATACATTATACGAACGGTATTACTTCTCAGCGGCCAGTTC	P1
oEC0085_F	CTCACAGGAGGACCCCACATGAAACCACTGCTGTATATCACCCC	P6
oEC0158_R	GCATACATTATACGAACGGTATTATCGAGCTACAGCCCCTTCC	P6
oEC0087_F	CTCACAGGAGGACCCCACATGCAGAATCGTTTACAATCAGGCG	P8
oEC0160_R	GCATACATTATACGAACGGTATCAGACTGTTTGCAAATTCCCCG	P8
oEC0090_F	CTCACAGGAGGACCCCACATGACGCATCGCCGGACCTC	P11
oEC0163_R	GCATACATTATACGAACGGTATCAGCGACGAGCCACTAACG	P11
oEC0092_F	CTCACAGGAGGACCCCACATGTCACCCTCTGATGTCCCC	P13
oEC0165_R	GCATACATTATACGAACGGTATCATTCTATTGCCAGTCTGCGCC	P13
oEC0094_F	CTCACAGGAGGACCCCACATGAACCGCCCCGCTGGTTC	P15
oEC0167_R	GCATACATTATACGAACGGTATCAGCAGCGTTGCATTCCATC	P15
oEC0095_F	CTCACAGGAGGACCCCACATGAACAGCCGACTCGAAACACAC	P16
oEC0168_R	GCATACATTATACGAACGGTACTACCTGACAATTGCCTTACGTTTG	P16
oEC0152_F	CTCACAGGAGGACCCCACATGAGAAACAGCAACCGCGGC	P19
oEC0171_R	GCATACATTATACGAACGGTATCACTGAGCCGATCCTACGG	P19
oEC0099_F	CTCACAGGAGGACCCCACATGCCCAACTTCACCACGCC	P20
oEC0172_R	GCATACATTATACGAACGGTATTACTCCGGTTTTGACGGTGC	P20
oEC0101_F	CTCACAGGAGGACCCCACATGGGAGCGCGCGCTATATTC	P22
oEC0174_R	GCATACATTATACGAACGGTATCACCGGGGTGCAGCCCAC	P22
oEC0087_F	CTCACAGGAGGACCCCACATGCAGAATCGTTTACAATCAGGCG	P23
oEC0160_R	GCATACATTATACGAACGGTATCAGACTGTTTGCAAATTCCCCG	P23
oEC0103_F	CTCACAGGAGGACCCCACATGTCCTGGATCATCCTGTTTTTCG	P24
oEC0176_R	GCATACATTATACGAACGGTATTAGCTGGCGCTGACTTTCAGG	P24
oEC0104_F	CTCACAGGAGGACCCCACATGAATCTGTATTGGGTCGTGTTATTAC	P25
oEC0177_R	GCATACATTATACGAACGGTATCAGGCCGGACCACTGGTC	P25
oEC0106_F	CTCACAGGAGGACCCCACATGCTGACCCTGAACTGGATTCC	P26
oEC0179_R	GCATACATTATACGAACGGTATCAGGCCAGCTTGAGCAGGC	P26
oEC0108_F	CTCACAGGAGGACCCCACATGAACGCGCTACGCGGCTG	P27
oEC0181_R	GCATACATTATACGAACGGTATCATGGTGCTTTCCTCGACGG	P27
oEC0109_F	CTCACAGGAGGACCCCACATGACCAACTATCTCTACCTCGCC	P28
oEC0182_R	GCATACATTATACGAACGGTATCAGTGCCCCGAAGCGCGG	P28
oEC0114_F	CTCACAGGAGGACCCCACATGAATGCCTATACCTACCTCGC	P30
oEC0187_R	GCATACATTATACGAACGGTATCAATGCCCAGCGGTCTTCG	P30
oEC0103_F	CTCACAGGAGGACCCCACATGTCCTGGATCATCCTGTTTTTCG	P31
oEC0188_R	GCATACATTATACGAACGGTACTAATGGCTCAGCTTGAGGCC	P31
oEC0118_F	CTCACAGGAGGACCCCACATGCGTTCCTGGATCTATCTGTTAC	P34
oEC0191_R	GCATACATTATACGAACGGTATCAGCATGTGACGGCCTCCC	P34
oEC0124_F	CTCACAGGAGGACCCCACATGACACCCTCAACGACGCC	P36
oEC0197_R	GCATACATTATACGAACGGTATCATGACAGACGGAGTAAAATCGC	P36
oEC0133_F	CTCACAGGAGGACCCCACATGCTCAAATCAGTTTTATATAAAAACTTC	P41
oEC0206_R	GCATACATTATACGAACGGTACTAAATAGGAAAGGGGCTTACC	P41
oEC0135_F	CTCACAGGAGGACCCCACATGCCGCTTTTTACCTCCTCTG	P43
oEC0208_R	GCATACATTATACGAACGGTATCACGATCTGGCAAACCATGTAC	P43
oEC0136_F	CTCACAGGAGGACCCCACATGTCGCTTGCTAAAGCCTCCC	P44
oEC0209_R	GCATACATTATACGAACGGTATCATGCTCGCCTACGCCAGAG	P44
oEC0140_F	CTCACAGGAGGACCCCACATGCTCGGCTCCGCCTTCTG	P48
oEC0213_R	GCATACATTATACGAACGGTATCAATCGGTGCGCCAGGGC	P48
oEC0148_F	CTCACAGGAGGACCCCACATGCATAACGATAAAGATCTCTCTACG	P56
oEC0222_R	GCATACATTATACGAACGGTATCATTGGCCAAATTGCATCTTATGTAG	P56
oEC0150_F	CTCACAGGAGGACCCCACATGGCGTGTGAACGGCTCGG	P58
oEC0224_R	GCATACATTATACGAACGGTATCAATTTCCGCGCTTGGCGTC	P58
oECB0009_R	CTCACAGGAGGACCCCACGTGAGCTTCCTTGTAGAAAATCAATTACTCG	P64
oECB0008_F	GCATACATTATACGAACGGTACTAGATAAGTAGGAACAACAACGTTTGGG	P64
	**Downstream amplicon**	
oTV34_F	TACCGTTCGTATAATGTATGC	
oEG1364_R	CTTGCATGCCTGCAGGTCGACGGCGTGTGGGATCGATGCTCAGG	
	**PCR on assembly product**	
oEG1345_F	GAAGACCAGCCCTGCTCCCAGCAG	
oEG1365_R	GTCGAGCCATTGCACGTCGTT	
	**Colony PCR**	
oEC1059_F	CCAGGACTTGCACCTTGAGCATG	
oEC332_R	CTCGATCATCGCCACAGCTTC	
	**GATC sequencing**	
EH120	CAACATGATGACACCGAGC	
EH366	CGACCACTTGATCACCACG	

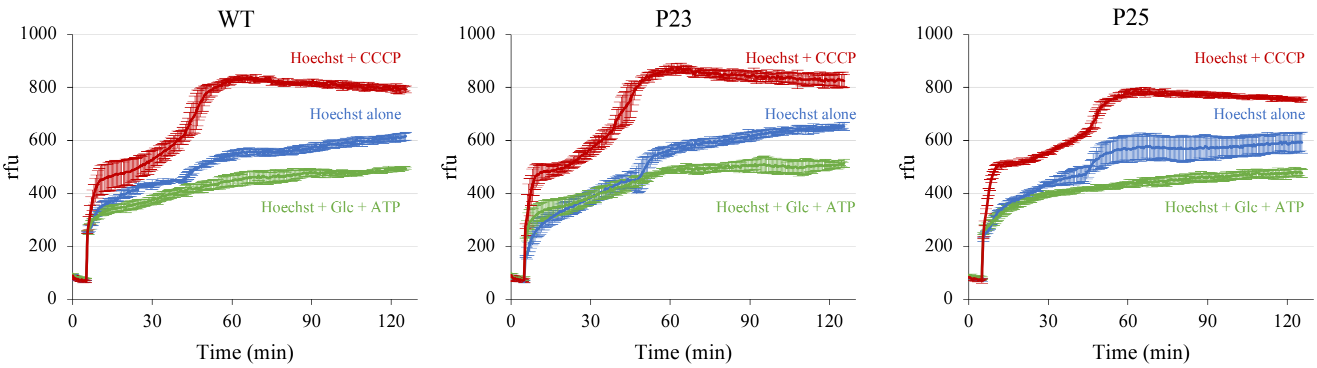

Supplementary Figure S3. Measurements of Hoechst 33342 fluorescence accumulation over time in the WT strain and P23 and P25 recombinant strains. The curves represent the mean values (with standard deviation) of the accumulation of Hoechst 33342 fluorescence over time in bacteria incubated with 2.5 µM Hoechst 33342 alone (blue curves), 2.5 mM Hoechst 33342 with 10 µM CCCP (red curves), or 50 mM glucose (Glc) and 1 mM ATP (green curves). For each value of the curve, the standard deviations are represented by vertical bars on the curves. The comparison of the curves is not significant in Student's t-test (data not shown). Abbreviations: rfu: relative fluorescence units, ns: not significant. All the results were obtained from biological triplicate.

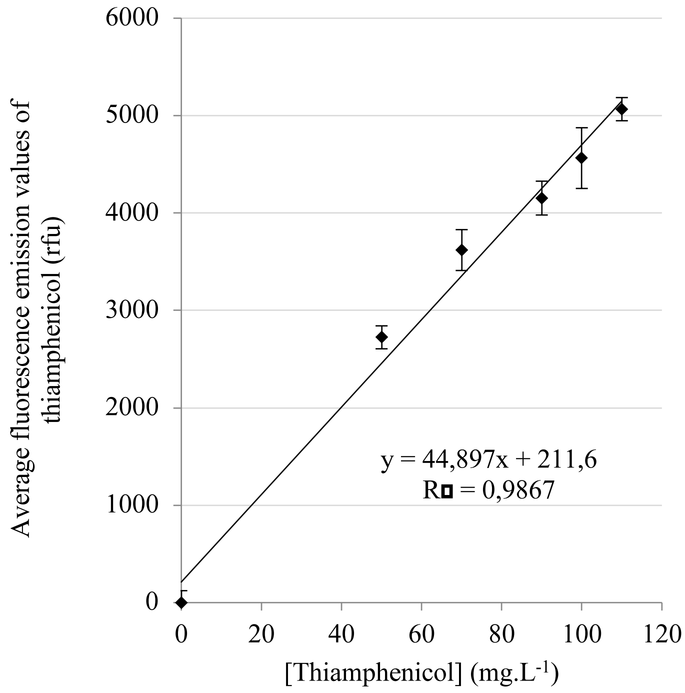

Supplementary Figure S4. Calibration curve of the thiamphenicol fluorescence signal. Measurements were obtained from control experiments performed without bacterial suspension in the same conditions as those used during the assay. The average values of the emission signals measured with increasing concentrations of thiamphenicol were reported in rfu (relative fluorescent unit). The linear correlation between the fluorescence emission signal and the concentration of thiamphenicol was validated by a corresponding Pearson's correlation coefficient of 0.9867. Four independent experiments were performed (with technical triplicates) and the means (± standard deviation) were presented.

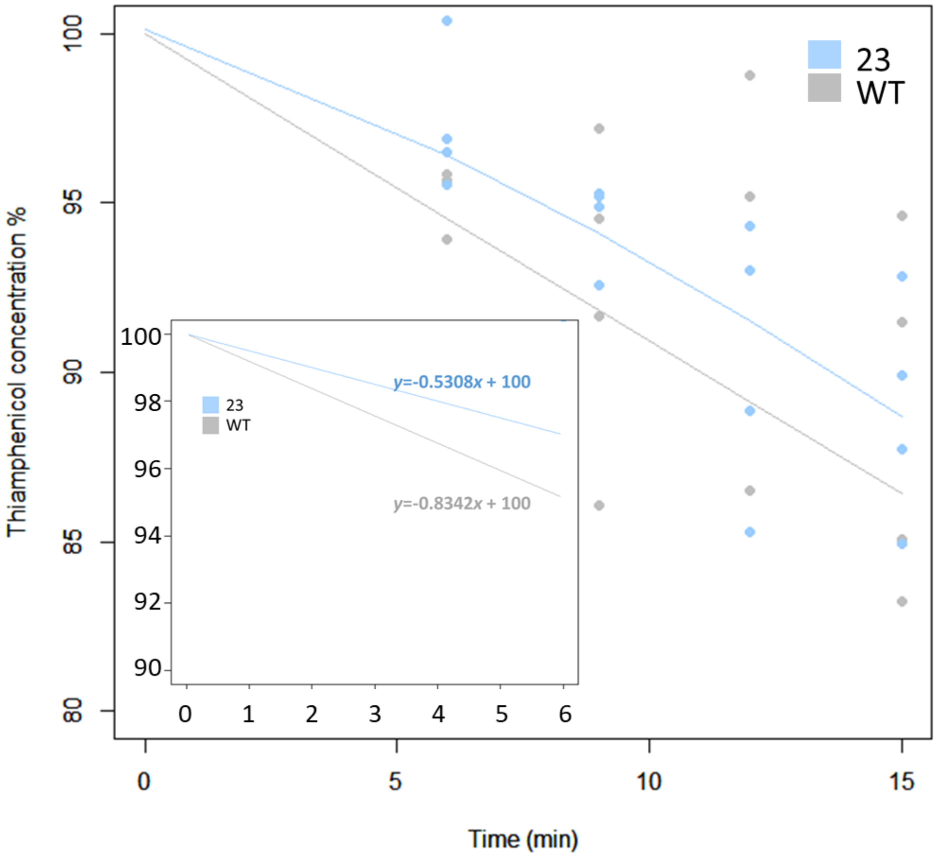

Supplementary Figure S5. Fitted curves of fluorescence intensities percentages of thiamphenicol (zoomed to 80-100 percent) measured over time in the extracellular environment during the incubation of WT (grey) and P23 (blue) strains. The inset presents the slopes of the curves obtained at the early incubation time (6 minutes) illustrating the ratio of 1.6 between the concentration measured in the extracellular medium of the P23 and WT strains.
